# Modern teaching for clinical education involving manual therapy: a 6-pillar approach

**DOI:** 10.1186/s12998-026-00646-y

**Published:** 2026-05-22

**Authors:** Kenneth J. Young, Roger Kerry, Cecilia Bergström, Sylwia Borowicz, Lisa J. Cary, Chad Cook, James Coughlan, David W. Evans, Vasileios Georgopoulos, Nathan Hutting, Edward Lee, Anna Maria Mazzieri, Chris McCarthy, Daniel Moore, Firas Mourad, Colette Ridehalgh, Clarice Tang, Nicholas Tripodi, Steven Vogel

**Affiliations:** 1 Victoria University, Melbourne, Australia; 2 School of Health Sciences, Queens Medical Centre, University of Nottingham, Nottingham, UK; 3 Unit of Obstetrics and Gynecology, Department of Clinical Sciences, Umeå University, Umeå, Sweden; 4 Polytechnic Institute Australia, Geelong, VIC 3220 Australia; 5 Department of Orthopaedics, Duke University, Durham, NC USA; 6 School of Sport, Exercise and Rehabilitation Sciences, University of Birmingham, Edgbaston, Birmingham, B15 2TT UK; 7 Injury, Recovery and Inflammation Sciences, School of Medicine, University of Nottingham, Nottingham, UK; 8 Department of Occupation and Health, School of Organization and Development, HAN University of Applied Sciences, Nijmegen, The Netherlands; 9 Centre for Pain Research, Health Sciences University, Bournemouth, UK; 10 The ST School, Exmouth, UK; 11 Department of Health Professions, Manchester Metropolitan University, Manchester, UK; 12 School of Health and Life Sciences, Teesside University, Middlesbrough, England, UK; 13 Department of Health, LUNEX University of Applied Sciences, 50, Avenue du Parc des Sports, 4671 Differdange, Luxembourg; 14 Luxembourg Health and Sport Sciences Research Institute A.S.B.L., 50, Avenue du Parc des Sports, 4671 Differdange, Luxembourg; 15 Facoltà Dipartimentale di Medicina e Chirurgia, Università Campus Bio-Medico di Roma, Rome, Italy; 16 School of Life Course and Population Sciences, Faculty of Life Sciences and Medicine, King’s College London, London, UK; 17 College of Sport, Health and Engineering, Victoria University, Melbourne, Australia; 18 College of Science, Health and Engineering and Institute for Health and Sport, Victoria University, Melbourne, Australia; 19 Centre for Osteopathic Research and Leadership (CORaL), UCO School of Osteopathy, Health Sciences University, London, UK

**Keywords:** Evidence‑based practice, Communication skills, Cultural safety, Manual therapy education, Physiotherapy, Osteopathy, Chiropractic, Soft‑tissue therapy, Professional identity, Epistemology, Axiology, Student-centred learning

## Abstract

**Background:**

Musculoskeletal (MSK) conditions remain a leading source of global disability, and manual therapy (MT) features in multimodal guideline concordant care; however, teaching in clinical programs that involve MT often remains technique-centric and insufficiently aligned with contemporary, person-centred teaching and practice. The present paper synthesizes literature and recent professional discourse to advance an educational framework that operationalizes modern clinical teaching across professions.

**Objective:**

To present a review-informed, consensus-driven, student centred framework for clinical education involving MT, designed to cultivate safe, effective, culturally responsive, and evidence-based graduates.

**Methods:**

Elements supporting a positive, student-centred learning experience were developed inductively by the lead author, then a narrative review of the literature was conducted on the elements, or pillars as they were designated. This was followed by iterative process to refine and further develop the ideas with a group of international educators, clinicians, and researchers across multiple professions. Consensus was achievedon the core elements of a modern clinical education framework.

**Results:**

The framework is anchored in basic sciences and clinical skills, and articulated through six teachable pillars: self-awareness, communication, cultural respect, epistemic humility, intellectual curiosity, and professional identity, delivered via high engagement, student-centred teaching. It provides practical curriculum guidance (crossyear learning outcomes, assessment strategies, and implementation vignettes) to embed person-centred communication, bias mitigation, cultural safety, evidence-based practice competencies, lifestyle medicine integration, and interprofessional collaboration. Together, the six pillars offer a novel approach to student-centred learning.

**Conclusions:**

A six-pillar, multi-profession approach offers actionable guidance for modern clinical education involving MT and supports the development of graduates prepared for contemporary MSK care: technically competent, evidence-based communicators who are culturally responsive and collaborative across healthcare teams.

## Background

Musculoskeletal (MSK) conditions remain a leading cause of global disability and health burden. Manual therapy (MT) continues to feature in clinical guidelines as part of multimodal care for these conditions [1–3]. In 2024, Kerry et al. (including much of the current author team) published “A modern way to teach and practice manual therapy,” proposing a framework for MT education and practice that moves away from the traditional principles of clinician-centred assessment, patho-anatomical reasoning, and technique specificity, and toward a humanistic, evidence-based model grounded in safety, comfort, and efficiency, contextualized by communication, therapeutic alliance, and person-centred care [4].

That paper stimulated international discussion and provided an aggregation of evidence for the way MT can be practiced and taught across physiotherapy, osteopathy, chiropractic, and soft-tissue therapy programs. However, its educational component focused primarily on operationalizing three practical dimensions (safety, comfort, efficiency) and three conceptual themes (communication, context, person-centred care) within teaching and clinical encounters. Building on this international momentum, it became clear that these professions are increasingly aligned on the need to not only modernize clinical practice, but also to reconsider how MTs are taught, conceptualized, and integrated within contemporary healthcare.

The current paper follows from the original by expanding on the educational element. It builds on the conceptual framework offered in the original paper and embraces a modern definition of MT [5]. It includes current approaches to holistic, person-centred care as deliverable by clinicians using MT. Ideas receiving improved and updated emphasis include education in self-awareness, communication mastery for clinical and interprofessional environments, and cultural respect and responsiveness.

The paper offers an approach that prioritises and fosters student-centred, active participation in the learning process, and provides curriculum guidance, assessment strategies, and implementation vignettes. In doing so, it offers learners a framework to apply foundational knowledge from their course learning and apply safe patient care. This approach aims to support programs in producing graduates who are not only technically competent and evidence-based skilled communicators but also culturally and contextually responsive collaborators, attributes increasingly recognized as central to quality MSK care and interprofessional practice [6–8].

Approaches to teaching, like approaches to clinical care, require continual updating as new evidence emerges supporting effective practices that promote student autonomy and improve student motivation. Rather than perceiving students as “empty vessels” into whom basic science knowledge (anatomy, physiology, etc.) and clinical skills are poured, a contemporary approach would seek to maximize each student’s potential. By helping students understand their own innate characteristics, they can be assisted in improving those characteristics that will help them acquire the knowledge and skills to serve the public to the best of their abilities while simultaneously building the elements, such as resilience and autonomy, that create a satisfying career.

Indeed, this move encourages students to take responsibility for their own learning. As a result, the teaching professional needs to “let go” of the familiar traditional approaches in order to promote this shift [9]. This transition highlights the importance of supporting students to become lifelong learners as successful professional graduates and future collaborative peers [9].

Practitioners who employ manual therapy should adopt a contemporary approach that incorporates person-centred care and self-management support as key elements, and it is therefore important that future educational standards in this field incorporate these components extensively [10]. Kerry et al. [4] highlight that historical teaching models continue to dominate curricula even though modern MT should be contextualised by therapeutic alliance, communication, and shared decision-making rather than technique mastery alone. Similarly, Keter et al. [11] emphasise that while person-centred care is increasingly recognised as essential in musculoskeletal practice, its integration into MT training remains limited and underdeveloped, indicating a clear need for frameworks that embed relational, contextual, and biopsychosocial competencies into teaching. This documented misalignment between outdated educational paradigms and contemporary evidence-based expectations substantiates the need for innovative approaches to modernise MT education and better prepare graduates for current clinical realities. Just as clinical practice should now be person (patient)-centred, so should teaching be student-centred.

A contemporary educational approach should incorporate a deep understanding of the importance of knowing “how we know what we know” (epistemology) and values and judgments (axiology) as professional clinicians and an examination of the values underpinning good practice. Indeed, “Learning axiology is important for understanding values, making ethical decisions, appreciating aesthetics, engaging in social and political discourse, and fostering personal and cultural development” [12].

Importantly, this approach does not imply abandoning the development of technical skills or the deliberate construction of clinician confidence in the safe and effective application of MT. On the contrary, technical proficiency remains essential, but it is framed within a wider clinical reasoning and person-centred communication process that enhances comfort, trust, and shared decision-making.

The aim of this paper is to propose a student-centred, literature-informed, consensus-driven educational framework for the teaching of health professionals who use manual therapy, designed to support safe, effective, culturally responsive, and evidence-based clinical practice across professions and educational contexts.

## Methods

The ideas for the approach outlined in this paper had their genesis during the development of a new chiropractic course at Victoria University in Melbourne, Australia. Major considerations included empowering students as reflective, autonomous learners and future clinicians/academics/researchers/policymakers with a drive for lifelong learning and satisfying professional identity. Developing a course with no pre-existing curriculum or staff allowed the opportunity to consider a new approach to teaching manual therapy. However, the concepts presented reflect the considerations of many educators across multiple institutions around the world as they develop new programmes and validate existing courses and are applicable across both higher education and vocational education sectors, for new or existing clinical programmes which include MT.

The approach aligns with the well-established pedagogical framework of Transformative Learning which remains fundamentally about helping students shift mindset and beliefs to then emerge with a sustainable identity [13, 14]. The acquired knowledge and skills will then be transferable and ensure a growth-mindset in their professional lives [15].

A multidisciplinary, international collaboration was developed to offer a wide variety of perspectives and allow for potentially wide application. All co-authors from the preceding paper (Kerry, et al.) were invited and some responded positively (CB, CC, DE, VG, NH, EL, AMM, CM, FM, CR, and SV) Other authors were invited through the lead author’s network because of their expertise in teaching and manual therapy (LC, SB, JC, DM, CT, and NT).

The six pillars were developed inductively at first, by the lead author. Evidence-based practice was initially a pillar, but then replaced with intellectual curiosity, making all pillars personal characteristics to be nurtured and developed, which would then support evidence-based practice. The meanings and interpretations of the foundation (basic science and clinical skills), pillars, and roof (evidence-based practice) were discussed with the first included co-authors (LC and RK), who provided ideas on elucidation and implementation.

The lead author then used his institution’s library search function, which aggregates articles from an extensive set of databases (Medline, PEDro, Cochrane, etc.) for information on each pillar in an educational context. Included documents consisted of literature reviews, primary research, policy documents, and editorials.

Subsequently, input from a broader spectrum of stakeholders was sought. A group of experienced, internationally based educators (Australia, UK, Europe, USA), clinicians, and researchers from across the spectrum of professions who utilise MT was convened. Perspectives were elicited through discussions in an iterative process using the Microsoft Word share function, which allows all authors to see the comments and changes of all authers. Feedback via questions and discussions was incorporated into further co-author discussions on the development of the framework. Consensus was achieved through repeated discussion of relevant elements. The consensus methodology was classed as educational development, which did not require ethical approval.

## Results

### Six pillars for teaching modern manual therapy

**Fig. 1:**
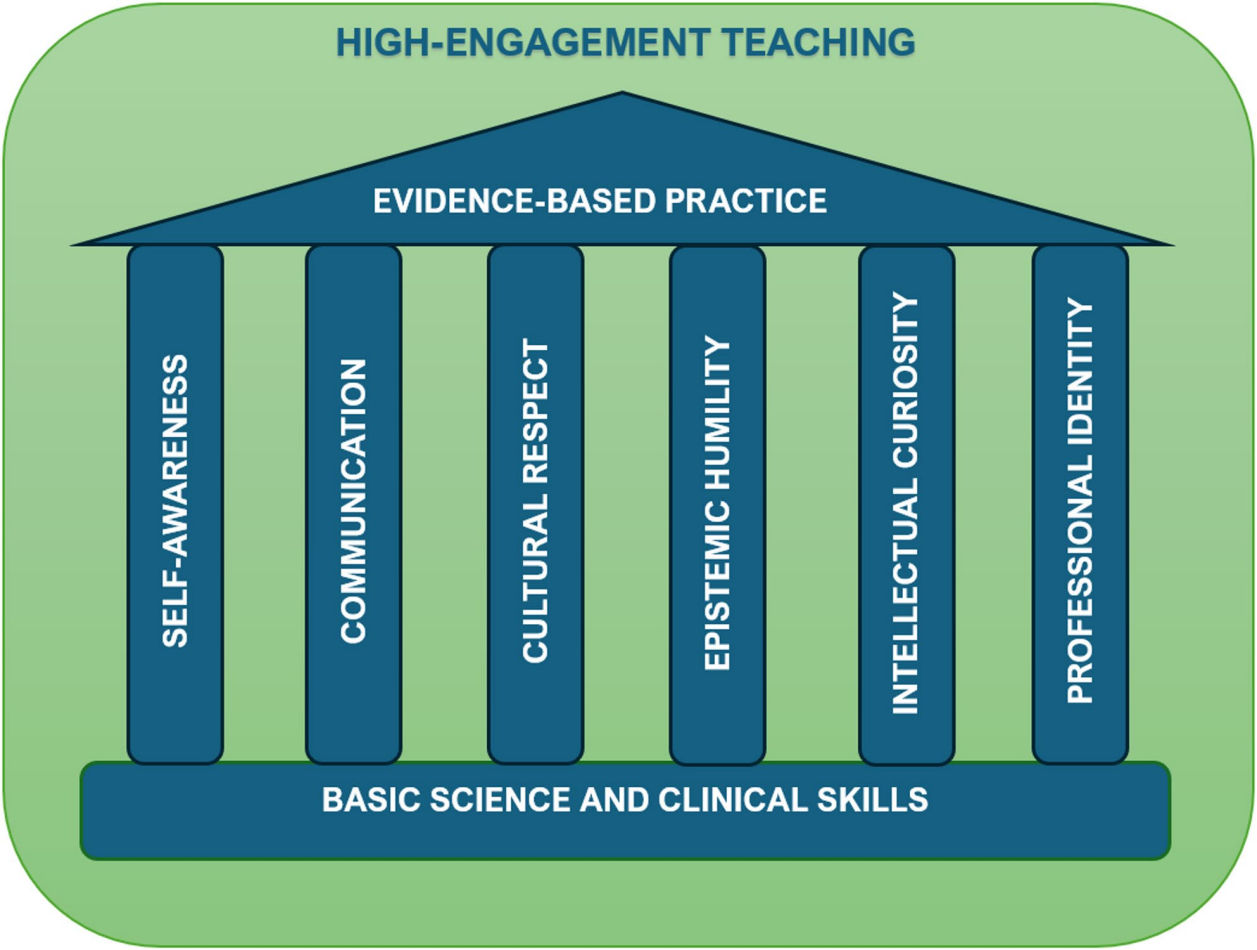
A modern approach to clinical teaching. The foundation of any clinical teaching is basic science and clinical skills. The 6 pillars represent personal characteristics that can be developed in this approach to support students achieving an ability to apply evidence-based practice. The surrounding and permeating green represents the high-engagement teaching methods through which all the elements of a clinical education may be delivered

The proposed framework for the teaching and learning of modern MT is built upon a foundation of basic sciences and clinical skills and held up by six ‘pillars’ that form the andragogical substance to support program development. These six pillars are: self-awareness, communication, cultural respect, epistemic humility (understanding the limits of one’s knowledge), intellectual curiosity, and professional identity. The foundation is basic science and clinical skills. The pillars support evidence-based practice (EBP), and the delivery is through high-engagement (student-centred) teaching, which is shown as permeating the entire academic environment (Fig. 1).

Each pillar represents an area of development to help the student achieve the fullest possible understanding of, and ability to apply, evidence-based practice. The focus of this approach is on the development of the (student) clinician as an evolving individual, rather than on topics taught. The topics in a clinical education environment are, of course, important, and should not be neglected when developing or revising a curriculum. However, students taught using this contemporary approach will develop the skills to engage meaningfully with clinical concepts and, importantly, other humans, including persons seeking healthcare, their carers and/or family members, and other health care professionals. Thus, students educated under this approach graduate with a clearer understanding of themselves, including their strengths and weaknesses, optimizing their ability to provide person-centred, collaborative, evidence-based care as required in the modern health care environment, while embracing life-long learning to adapt and thrive in an ever-evolving field.

### Pillar 1: self‑awareness

Incorporating self-awareness, including bias mitigation, into clinical education is essential for fostering equitable and person-centred care and for underpinning the development of professional identity [16]. Cognitive and implicit biases can influence clinical reasoning and treatment decisions, often unconsciously shaping interactions with patients [17–19]. Educational strategies such as reflective practice, structured self-assessment, and multicultural competency frameworks have been shown to reduce bias and improve decision-making in health professions curricula [20–22].

Encouraging students to engage in guided reflection or personal therapy can enhance intrapersonal awareness, which correlates with improved therapeutic relationships and outcomes [23]. However, reflection without recognition of the knowledge gap or of the experience of uncertainty, may reinforce previous assumptions. Purposeful and mindful exposure to what Mezirow (1994) calls disorienting dilemmas [24], in the form of ethically challenging and complex clinical presentations can bring necessary disruption to those beliefs and bias, providing opportunity for shift in understanding. It is in this liminal space where the student can learn practical strategies to navigate complexity [25].

Integrating these approaches into MT programs supports the broader goal of preparing practitioners for varied clinical settings while reducing the risks associated with diagnostic momentum and clinician bias. Personality and empathy assessments provide practical tools for embedding self-awareness into training. Personality evaluation instruments such as the NEO-FFI or Big Five Inventory, combined with empathy measures like the Jefferson Scale of Physician Empathy and the Interpersonal Reactivity Index, help students understand how traits such as agreeableness and openness influence empathic engagement [26, 27]. Similarly, behavior change questionnaires based on the Transtheoretical Model [28], Theory of Planned Behavior [29], ASE model [30], or I-Change model [31] can support goal-setting and readiness assessment for professional growth and a more holistic approach to patient management in clinical practice [32, 33]. These tools not only facilitate personalized learning but also provide educators with data to tailor interventions that enhance cultural humility and reduce implicit bias. Embedding such assessments within clinical curricula promotes reflective practice and equips future clinicians with strategies to navigate complex interpersonal dynamics in patient care.

Reflective learning is a critical approach in health care education that fosters deep learning and professional growth. By encouraging students to critically analyze their experiences, reflective practice enhances self-awareness, clinical reasoning, and the ability to integrate theory with practice. Research shows that reflective activities such as writing, peer feedback discussion, and creative methods help students process emotions, develop empathy, and improve communication skills, which are essential for person-centred care [34, 35]. Furthermore, reflective learning promotes metacognition and lifelong learning, enabling future practitioners to adapt to complex clinical environments and continuously improve their practice [36, 37]. Evidence also suggests that reflection strengthens professional identity and supports the development of compassionate care, making it a cornerstone for both academic success, clinical competence [38, 39] and may reduce rates of professional burnout [40].

### Pillar 2: Communication

Developing verbal and non-verbal communication skills is fundamental to the education of manual therapists, both for clinical and interprofessional situations [41, 42], as is having narrative competencies which enables therapists to understand and interpret patients’ perspectives and respond to their stories [43–46]. These competencies underpin effective clinical interactions and therapeutic alliances [46]. The Calgary-Cambridge approach is a useful and commonly used structure for clinical encounters [47].

Verbal communication strategies like the OARS (open question, affirmation, reflection and summary), “five cards”, or ICE (patient’s Ideas, Concerns, and Expectations) models enhance patient engagement and trust [48–50]. Non-verbal elements, including eye contact, posture, facial expressions, and proxemics, complement spoken language and convey empathy, attentiveness, and professionalism [51, 52]. Research in allied health education emphasizes that congruence between verbal and non-verbal cues is critical for reducing patient anxiety and improving satisfaction [53, 54] as well as for improving equity [55].

Good communication skills help build the therapeutic alliance between practitioner and patient, which improves safety and clinical outcomes [56]. Clinicians, including those who employ MT, may contribute to suicide prevention by employing advanced communication strategies to identify indicators of psychological distress, respond with professional empathy, facilitate timely referral to appropriate mental health services, and document concerns with sensitivity, recognizing that these actions can play an important role in reducing risk [57, 58].

Incorporating structured training, such as role-play with standardized patients and video feedback, has been shown to significantly improve students’ ability to integrate these skills into clinical practice [59]. Educating students about shared-decision making, such as involving families in discussions about care plans helps ensure that emotional, cultural, and social needs are addressed, and contributes to holistic care and improved well-being for both patients and caregivers [60, 61].

Communication skills also play a pivotal role in interprofessional collaboration, where clarity and respect are essential for patient safety, team, and interdisciplinary efficiency. Interprofessional Education (IPE) initiatives demonstrate that joint training in communication fosters shared decision-making, reduces clinical errors, and enhances teamwork across disciplines [62, 63]. Non-verbal behaviors—such as open body language and active listening—are particularly important in multidisciplinary settings, where subtle cues can influence perceptions of competence and collegiality [64]. Evidence suggests that simulation-based interprofessional communication training improves attitudes toward collaboration and error disclosure, reinforcing the need for experiential learning approaches in MT curricula [65]. Embedding these strategies ensures graduates are prepared not only for patient-centred care but also for effective participation in complex healthcare teams.

### Pillar 3: Cultural respect

Cultural respect can be defined as the recognition, protection and continued advancement of the inherent rights, cultures and traditions of a particular culture. Cultural respect and responsiveness are integral to safe, effective, and equitable care. The clinical encounter is inherently relational and value-laden, as patient-centred care, evidence-based practice, and value-based healthcare all position patient values at the core of high-quality clinical practice. Consequently, enhancing students’ awareness and understanding of patient values represents a fundamental component of contemporary healthcare education and delivery [66]. Clinical understanding arises through the integration of the patient’s narrative, emotions, beliefs, and prior experiences with those of the practictioner. This reciprocal process generates an intersubjective clinical space in which shared meaning, respect, and interpretive sensitivity are essential for developing a coherent, clinically reasoned understanding to guide decision making [67]. Contemporary international frameworks for health equity emphasise the provision of a culturally safe environment through delivery of culturally responsive practice [67]. Cultural safety reflection tools [68, 69] and equity-focused metrics [70, 71] can help understanding of these concepts at a granular level.

Advocating for care that is effective, equitable, understandable, and respectful of patients' diverse cultural identities, health beliefs, preferred languages, and health literacy needs, as experienced and defined by patients themselves aligns to the WHO’s Integrated People‑Centred Health Services framework which centres care around people’s needs and preferences [72]. In Australia, health professional accreditation bodies such as the Chiropractic Board of Australia, Osteopathy Board of Australia, and the Australian Physiotherapy Council, [73] embed cultural safety and anti‑racism expectations into Practice Threshold Standards. State frameworks and training (e.g., Victoria’s cultural safety framework [74] and VACCHO programmes [75]) offer practical pathways. Likewise in the United Kingdom, ensuring culturally appropriate communication is identified as a crucial element of patient safety in addressing health inequalities [76].

Local community voices are important for curriculum development and can improve research collaborations [77, 78]. Students should understand the importance of language access to health care equity [79, 80] and how applying trauma‑informed principles improves outcomes [81, 82].

### Pillar 4: Epistemic humility

Epistemic humility involves an awareness of the uncertainty and fallability of claims about knowledge. It requires the sustained application of ethical principles to move aross the inherent tension between evidence-based knowledge and the contextual realities [83]. Acknowledging that one’s beliefs may be wrong, incomplete, or biased, and that openess to alternative perspectives, ways of understanding or evidence may offer valuable insights. Professional histories may be juxtaposed with the evolution of scientific norms (falsifiability, study replication, peer review) to build epistemic humility [84–86]. This humility can sit alongside a fundamental ability to identify and appraise contemporary guidelines to highlight where robust evidence supports therapeutic interventions for specific clinical indications mapped to diagnoses. Healthcare dogma and pseudoscience are increasing threats to the learning of best practice across professions [87, 88]. Teaching anti-dogma and fostering understanding of pseudoscience in health professions education is critical for developing clinicians who can navigate uncertainty and resist unsubstantiated claims. Educational strategies that address medical misinformation and credentialed arrogance underscore the importance of humility and evidence-based reasoning in countering dogma [89–91].

The educational aim under this pillar is for learners to explore areas where uncertainty persists and develop comfort with epistemic flexibility and clinical uncertainty [92–96]. The promotion of strategies may then be offered on how to proceed with care using the best available information, maintaining differential diagnoses and continually re-evaluating progress is also recommended. [97–99].

### Pillar 5: Intellectual curiosity

Intellectual curiosity is particularly important to developing an evidence-based approach to practice. Programs can train the five steps of Evidence Based Practice (ask, acquire, appraise, apply, assess) [100, 101] and map competencies to consensus frameworks [102, 103]. Utilizing a variety of teaching strategies (e.g. structured journal clubs, case‑based learning, simulation) and activities will foster openness and intellectual curiosity [104].

Fostering intellectual curiosity in healthcare students is vital for developing adaptive, lifelong learners who can thrive in complex clinical environments. Curiosity drives students to identify knowledge gaps, engage in self-directed learning, and explore innovative solutions, which enhances critical thinking and problem-solving skills essential for safe and effective patient care [105]. Studies show that curiosity not only improves academic performance but also strengthens professional identity and empathy, enabling practitioners to build deeper patient relationships and deliver person-centred care [106]. Moreover, curiosity is linked to resilience and well-being, helping future clinicians maintain motivation and adaptability in the face of evolving healthcare challenges [107].

Lifelong learning promotes continuous skill development, critical thinking, and evidence-based practice, enabling future clinicians to stay current with advances in technology, treatments, and patient care standards [108, 109]. Research highlights that ongoing learning enhances clinical judgment, problem-solving, and professional identity, while also improving patient safety and outcomes through the application of updated knowledge and practices [110]. Moreover, cultivating a growth mindset and self-directed learning habits during training equips students to engage in reflective practice and adapt to new challenges throughout their careers [111].

### Pillar 6: Professional identity

It is important to help students develop their professional identity as it can help people engage more meaningfully and feel more satisfied with their work [112–116]. In the absence of a satisfying professional identity, people will seek it out, sometimes turning to traditional ideologies or financially oriented practice management schemes [117–119]. Although manual therapies all have unique histories and cultures, they share important features that form the basis for an evidence-based identity (as opposed to an identity based on the traditional features identified previously by Kerry, et al.—clinician-centred assessment, patho-anatomical reasoning, and technique specificity [4]). They are all MSK specialist professions whose scope of practice includes a variety of therapeutic interventions, including movement, and behaviour‑centred care, manual techniques like joint and soft tissue mobilization and manipulation, and collaboration across disciplines. The behaviour centred aspects are neatly encapsulated as congruent with Lifestyle Medicine (physical activity, sleep, social connections, smoking, alcohol and substance reduction, healthy nutrition, and environmental factors).

Integral to the development of professional identity is an understanding of the need for self-care, which is essential for maintaining well-being, preventing burnout, and ensuring high-quality person-centred care [120, 121]. Health care students and professionals face intense emotional, cognitive, and physical demands that can lead to stress, compassion fatigue, and professional impairment if not proactively managed [122]. Engaging in self-care practices such as mindfulness, physical activity, peer support, and reflective journaling has been shown to enhance resilience, emotional regulation, and clinical performance [123]. Moreover, integrating self-care education into health curricula improves students’ coping strategies and fosters a culture of wellness that extends into professional life [124].

Professional identity is also not a static concept. It evolves in response to the changing nature of patient needs and societal expectations. As healthcare becomes increasingly patient-centred, diverse and technology driven, clinicians must adapt their sense of identity to remain relevant and effective. By fostering an identify throughout any pre-professional clinical training that is grounded in adaptability and lifelong learning, educators prepare students to meet future challenges.

### Proposed educational model: an example of potential practical application

Across all years: Integrate local community voices; adopt cultural safety reflection tools; ensure language access; apply trauma‑informed principles; and evaluate with equity‑focused metrics. A 4/5 year curriculum used as an example. Pacing adaptable to other programme lengths (e.g. 2–3 years).

Year 1 (Foundations): Self-awareness inclusive of scope of practice and development of professional identity, EBHC steps; professional histories in physiotherapy/osteopathy/chiropractic/soft‑tissue therapy; science vs dogma; dual‑process reasoning and biases; introductory communication micro‑skills; introduction to cultural awareness; learning about other health professionals.

Year 2 (Skills & evidence): literature searching and evidence hierarchy; appraisal labs (RCTs, systematic reviews, guidelines, with material on epistemic humility); structured journal clubs; Calgary‑Cambridge‑based clinical assessments; verbal and non‑verbal communication and teleconsultation training. Learning from other health professionals.

Year 3 (Integration): Lifestyle Medicine strategies; guideline mapping; care‑pathway design; cultural safety projects co‑designed with local communities; learning together with other health professionals where students from different disciplines come together to share professional-specific knowledge and improve communication skills and interprofessional collaborations.

Year 4/5 (Critical Analysis and Clinical Reasoning): Clinical scenario-based assessments (shared decision‑making; exercise prescription; indications/contraindications for MT); suicide prevention strategies; reflective synthesis on identity, communication growth, and cultural responsiveness. Developed shared decision-making skills, conflict management in collaboration with other health professionals.

### Implementation vignettes (illustrative)

Vignette A: (Foundation) Foundation unit across professions: Mixed cohorts of physiotherapy, osteopathy, chiropractic and soft‑tissue therapy students complete shared communication labs (Calgary‑Cambridge), IPE collaborative teamwork simulations, and cultural safety workshops co‑facilitated by community partners; assessments include OSCEs or vivas with communication/global ratings and reflective journals mapped to EBHC and cultural responsiveness.

Vignette B: (Skills and Integration) Teaching clinic redesign: Intake scripts screen for red flags and psychosocial risk; imaging avoided without indication; care plans foreground education, exercise, and Lifestyle Medicine components; clinicians document consent and boundaries for touch; monthly “EBHC huddles” review cases and communication metrics (warmth/listening; teach‑back).

Vignette C: (Assessment & Identity) ‘Contemporary Challenges’ critical discussion sessions—e.g. what does ‘non-specific’ mean for professional identity? ‘Leadership’ critical workshops—how can devolved leadership facilitate de-implementation of out-dated theory and skills? ‘Quality Improvement’ critical workshops—how can students engage with educational processes to re-conceptulise MT learning.

## Discussion

This paper proposes a curriculum approach for clinical education involving MT that operationalizes six pillars within an environment of high-engagement teaching. The pillars are self-awareness, communication, cultural respect, epistemic humility, intellectual curiosity, and professional identity. The intent is to prepare students for contemporary MSK practice that is safe, effective, and congruent with modern health care delivery, equipping graduates for interprofessional collaboration and culturally diverse clinical contexts. This focus aligns closely with the directions proposed by Kerry et al. [4], whose framework called for a shift away from mechanistic, technique-specific teaching toward an educational model grounded in communication, contextual awareness, and person-centred practice; the six pillars advanced in the present paper extend and operationalize these foundations by making them explicitly teachable components of clinical education.

Deploying the approach in this paper means that units of learning cannot be siloed and the inevitable teaching inertia that develops in more longstanding courses must be overcome. To maintain competency in an ever changing sector, lifelong learning has long been required for health care professionals, so in a similar way, teachers as well as students need to be open to change, collaboration, and new ideas to maximize effectiveness as academics or future clinicians.

Modern teaching must involve high levels of engagement, integrating and upgrading traditional didactic approaches in large lecture halls. Where large volume teaching modes are used, they should include participatory activity from learners and in parallel smaller student number learning opportunities to integrate, reflect and apply material in high engagement contexts. High-engagement teaching fosters active participation, deeper learning, and improved retention compared to passive lecture-based methods. Evidence from health professions education and adult learning research consistently shows that active learning strategies, such as problem-based learning (PBL), simulation-based training, and collaborative exercises, enhance critical thinking, motivation, and knowledge transfer [125].

Adults, like university students, learn best when content is relevant, experiential, and connected to real-world practice, aligning with Knowles’ principles of andragogy that emphasize autonomy and problem-solving [126]. Practical approaches include simulation-based learning, which provides realistic clinical scenarios for skill application in a risk-free environment, PBL and TBL, where learners tackle authentic cases to develop reasoning and teamwork skills [127].

Other high-engagement strategies include microlearning, that is, delivering concise, focused content during natural breaks [128, 129], and structured debriefs to consolidate learning and encourage reflection [130, 131]. Incorporating visual tools, interactive technology, and collaborative projects further supports engagement by making learning dynamic and personalized [132, 133]. Any curriculum should be developed strategically, ensuring that key concepts such as informed consent and reflective practice are scaffolded throughout the course. Detailed assessment rubrics for key concepts should be developed with rigour and detail appropriate to the stage of learning in the course and linked to learning outcomes. These methods not only improve academic outcomes but also prepare learners for the demands of real-world healthcare environments where adaptability, critical thinking, and effective communication are core expectations of industry and employers. This approach to clinical education responds to the need for reform in MT education, moving away from traditional, technique-centric paradigms toward models emphasizing humanistic care, therapeutic alliance, and evidence integration [4]. Furthermore, it reflects broader trends in health professions education that prioritize communication, cultural safety, and reflective practice as core competencies for safe and equitable care [134].

Self-awareness, encompassing general emotional intelligence, is essential to full development as a health care provider but has not as yet been prominent in curricula [135]. Yet it would seem to be essential to understand how to relate to diverse patients and other health professionals, as well as for self-care [136]. Self-awareness can also improve career development. Some individuals complete clinical courses then discover that front-line clinical practice is not where their strengths or preferences lie [137]. But a clinical background is useful for a variety of careers, including in research, administration, or governance [138]. Understanding one’s self early can help focus educational opportunities to maximize professional success and satisfaction [137, 139].

Embedding structured reflection and bias mitigation strategies addresses well-documented risks of cognitive and implicit bias in clinical decision-making [16]. Evidence suggests that interventions such as guided reflection, personality and empathy assessments, and multicultural competency frameworks improve diagnostic accuracy and patient trust [140]. These strategies also align with international recommendations for bias training in health curricula to reduce disparities in care [141].

Communication remains a cornerstone of therapeutic alliance and patient safety. Frameworks such as Calgary-Cambridge and OARS have demonstrated efficacy in improving patient engagement and satisfaction [59]. Non-verbal congruence further reduces anxiety and fosters trust [142]. Interprofessional Education (IPE) initiatives confirm that joint communication training enhances teamwork and reduces clinical errors [143]. Incorporating simulation-based learning, clincial placement education and video feedback into curricula strengthens skill acquisition and transfer to practice [144, 145].

Cultural safety is now embedded in regulatory standards and global frameworks for person-centred care [146]. Evidence from umbrella reviews shows that cultural competency education improves provider attitudes and knowledge, though patient-level outcomes remain under-researched [134]. Practical strategies, including community engagement, language access, trauma-informed care, are essential for reducing inequities in MSK health, particularly among racialized and Indigenous populations who experience disproportionate burden and poorer outcomes [147, 148].

Teaching epistemic humility alongside EBP skills fosters critical thinking and diagnostic openness, mitigating risks of dogma and fostering flexibility of thinking [84, 149]. Longitudinal studies in physical therapy education show that EBP confidence and behaviors improve significantly with structured curricular integration, particularly after classroom instruction and early clinical exposure [150]. This supports the inclusion of scaffolded EBP training across the curriculum. By embedding strategies that cultivate intellectual curiosity, such as reflective practice, active questioning, and innovative learning methods, educators can prepare students for continuous growth and excellence in clinical practice [151, 152].

Professional identity formation is linked to career satisfaction and ethical practice [116]. Without a coherent identity, graduates may gravitate toward outdated ideologies or commercially driven models [153]. Positioning manual therapists as MSK specialists within a biopsychosocial and lifestyle medicine framework reinforces an evidence-based identity aligned with global health priorities [154]. Lifestyle Medicine competencies (physical activity, sleep, nutrition, social connection) are increasingly recognized as integral to conservative MSK care [155, 156]. Although this approach was developed for a manual therapy educational program, its application is not limited to manual therapy, and we think that the approach outlined here is broadly applicable in tertiary education.

### Limitations and future research

This model synthesizes existing evidence and standards but does not present empirical outcome data. Future research should prospectively evaluate competency attainment (including communication and cultural responsiveness), behavioural endpoints (appropriate imaging; guideline‑concordant first‑line care), interprofessional collaboration, and patient‑reported outcomes. Further, the recommendations presented in this paper face notable challenges in their implementation. First, the development was anchored to a pre-existing five-year, continuous study program. However, degree structures and regulatory environments vary widely across healthcare professionals who use MT. This variability complicates the direct transferability of the proposed framework and would require conscious application at varying periods in the training program. Second, the integration of these recommendations into existing curricula with the appropriate degree of fidelity will require significant time and may necessitate the replacement of established training paradigms. Such changes are particularly difficult to enact in the current climate of curriculum creep [157], a phenomenon increasingly observed in healthcare education and one that has already posed challenges in the training of manual therapists [158, 159]. In addition, there are issues of change management, resourcing, staff development.

## Conclusion

A six‑pillar, multi‑profession approach to education can help physiotherapy, osteopathy, chiropractic, and soft‑tissue therapy programs produce collaborative, culturally responsive clinicians who provide guideline‑concordant, MSK-focused care with a whole-person perspective. Together, the pillars create a novel, student-centred perspective and include self-awareness, communication, cultural respect, epistemic humility, intellectual curiosity, and professional identity. These six concepts, delivered through high-engagement teaching, allow effective delivery and uptake of basic sciences and clinical skills, leading to entry-level professionals, ready for evidence-based practice in the modern health care environment. Although this approach was developed for a manual therapy educational program, its application is not limited to manual therapy, and is broadly applicable in tertiary education.

## Data Availability

No datasets were generated or analysed during the current study.
